# Transverse Sinus Stenosis in Refractory Chronic Headache Patients: An Observational Study

**DOI:** 10.3389/fneur.2019.01287

**Published:** 2019-12-12

**Authors:** Valentina Favoni, Giulia Pierangeli, Luigi Cirillo, Francesco Toni, Samir Abu-Rumeileh, Chiara La Morgia, Monica Messia, Raffaele Agati, Pietro Cortelli, Sabina Cevoli

**Affiliations:** ^1^IRCCS Istituto delle Scienze Neurologiche di Bologna, Bologna, Italy; ^2^Department of Biomedical and Neuromotor Sciences, Alma Mater Studiorum—University of Bologna, Bologna, Italy; ^3^Neuroradiology Department, IRCCS Istituto delle Scienze Neurologiche di Bologna, Bologna, Italy

**Keywords:** chronic headache, refractory headache, MRI, sinus stenosis, idiopathic intracranial hypertension

## Abstract

**Background:** Transverse sinus stenosis is a common brain MRI finding in chronic migraine (CM) and chronic tension-type headache (CTTH) patients in clinical practice; however, its clinical and diagnostic role is unclear. The aim of the study is to determine the frequency of transverse sinus stenosis in these headache patients resistant to preventive treatments and to verify whether this is a useful finding for identifying patients with intracranial hypertension.

**Methods:** This is an observational study. Patients with refractory CM and CTTH underwent a 3T-magnetic resonance venography (MRV) before cerebrospinal fluid (CSF) opening pressure measurement. Transverse sinus stenosis was determined using the combined conduit score. Patients with opening pressure >200 repeated MRV study 1 month after CSF withdrawal to evaluate changes in neuroimaging findings.

**Results:** We analyzed MRV studies of 40 patients (32 F, 8 M; mean age, 49.4 ± 10.8; mean body mass index, 26.7 ± 6.4; 39 CM and 1 CTTH with concomitant episodic migraine). Nineteen cases (47.5%) had evidence of transverse sinus stenosis: bilateral in seven patients (17.5%) and unilateral in 12 cases (30%). No statistically significant differences in transverse sinus stenosis distribution were found between patients with opening pressure <200 mmH_2_O and those with opening pressure >200 mmH_2_O. On Spearman bivariate test, there was no correlation between opening pressure and combined conduit score. No changes in neuroimaging findings were found 1 month after CSF withdrawal.

**Conclusion:** Transverse sinus stenosis is a frequent radiological finding (47.5%) in CM and CTTH patients refractory to preventive treatments. However, this finding is not suggestive of intracranial hypertension. Whether transverse sinus stenosis may be a possible risk factor for chronic headache or a comorbidity needs to be evaluated in larger epidemiological studies.

## Introduction

Transverse sinus stenosis is a common brain MRI finding in chronic migraine and chronic tension-type headache patients in clinical practice, although a few studies evaluated it systematically (1–4). Previously reported transverse sinus stenosis prevalence ranges from 9% in a series of chronic tension-type patients to 92.8% in 44 refractory chronic migraine patients (1, 3). The importance of investigating the presence of transverse sinus stenosis in these patients, especially when refractory to medical treatments, arises from the need to rule out a condition of idiopathic intracranial hypertension without papilledema (IIHWOP). Headache attributed to IIHWOP may mimic chronic migraine or chronic tension-type headache, and the discrimination can be clinically difficult (5, 6). The diagnosis of IIHWOP can be suggested not only by elevated cerebrospinal fluid (CSF) opening pressure but also by the presence of at least three of the following neuroimaging findings: empty sella, distention of the perioptic subarachnoid space with or without a tortuous optic nerve, flattening of the posterior sclerae, and transverse venous sinus stenosis (Table 1) (7). Among these radiological features, transverse sinus stenosis is accepted as the most sensitive hallmark of the diagnosis, having a sensitivity of 84.4% [95% confidence interval (95% CI), 65.9–93.9%] and a specificity of 94.9% (95% CI, 91.7–96.9%) (8). Although, a possible bidirectional correlation between transverse venous sinus stenosis and IIHWOP has been previously reported (9), the role of transverse sinus stenosis in headache patients is still controversial. Moreover, transverse sinus stenosis may also be found in about one-third of the general population (10, 11). Indeed, 31% of 100 people with normal MR imaging findings had magnetic resonance venography (MRV) evidence of a transverse flow gap (10). More recently, using CT angiography, the prevalence of transverse sinus stenosis was 38% in 355 consecutive “healthy” subjects (11).

**Table 1:** Diagnostic criteria for pseudotumor cerebri syndrome (7).

**1. Required for diagnosis of pseudotumor cerebri syndrome^a^**
Papilloedema Normal neurologic examination except for cranial nerve abnormalities Neuroimaging: normal brain parenchyma without evidence of hydrocephalus, mass, or structural lesion and no abnormal meningeal enhancement on MRI, with and without gadolinium, for typical patients (female and obese), and MRI, with and without gadolinium, and magnetic resonance venography for others; if MRI is unavailable or contraindicated, contrast-enhanced CT may be used Normal CSF composition Elevated lumbar puncture opening pressure [≥250 mm CSF in adults and ≥280 mm CSF in children (250 mm CSF if the child is not sedated and not obese)] in a properly performed lumbar puncture
**2. Diagnosis of pseudotumor cerebri syndrome without papilloedema**
In the absence of papilloedema, a diagnosis of pseudotumor cerebri syndrome can be made if B–E from above are satisfied, and in addition, the patient has a unilateral or bilateral abducens nerve palsy. In the absence of papilloedema or sixth nerve palsy, a diagnosis of pseudotumor cerebri syndrome can be suggested but not made if B–E from above are satisfied, and in addition, at least three of the following neuroimaging criteria are satisfied: Empty sella Flattening of the posterior aspect of the globe Distention of the perioptic subarachnoid space with or without a tortuous optic nerve Transverse venous sinus stenosis

a *A diagnosis of pseudotumor cerebri syndrome is definite if the patient fulfills criteria A–E. The diagnosis is considered probable if criteria A–D are met but the measured cerebrospinal fluid (CSF) pressure is lower than specified for a definite diagnosis*.

The aim of this study was to clarify the clinical frequency and diagnostic role of sinus stenosis on MRV to identify patients with intracranial hypertension among chronic migraine and chronic tension-type headache patients resistant to prophylactic therapies.

## Materials and Methods

### Outcome Measures

The primary endpoint of the study was to analyze the frequency of transverse sinus stenosis in a series of consecutive refractory chronic headache (chronic migraine and chronic tension-type headache) patients.

The secondary endpoint was to evaluate the correlation between transverse sinus stenosis and CSF opening pressure to identify patients with intracranial hypertension and to evaluate changes in transverse sinus stenosis after CSF withdrawal.

### Standard Protocol Approvals and Patient Consents

This prospective study was conducted in agreement with principles of good clinical practice, and the study protocol was approved by the Local Ethics Committee of the local health service of Bologna, Italy (no. 12017/CE). All patients gave their written informed consent to study participation.

### Participants

Forty patients with refractory chronic headache (chronic migraine and chronic tension-type headache) prospectively underwent MRV at the tertiary Headache Center of IRCCS Institute of Neurological Sciences of Bologna, Italy, from September 2013 to February 2016, as part of a previous study (12). Diagnosis of the type of headache was established according to the International Classification of Headache Disorders-3 beta version criteria and also confirmed according the new International Classification of Headache Disorders-3 version criteria (13, 14). Refractoriness was defined as the failure of at least three trials of preventive therapies at adequate doses and at least one detoxification attempt in case of medication overuse. Inadequate response was defined as absence of clinically meaningful improvement after at least 3 months of therapy at a stable dose considered appropriate for migraine or tension-type prevention according to accepted guidelines (15, 16). As previously reported, detailed information about the clinical features of headache and associated symptoms potential-related comorbidities and concomitant medications were collected by face-to-face structured interviews (12).

### Study Protocol

In this prospective study, each patient underwent MRV before CSF opening pressure measurement. Complete details of the lumbar puncture procedure, and results were described elsewhere in detail (12). Briefly, according to previous findings that suggest that CSF withdrawal may result in a sustained remission of chronic migraine, we performed CSF withdrawal in patients with opening pressure values above 200 mmH_2_O: intracranial pressure measurements were repeated every 2 ml of extracted CSF, up to ~100 mmH_2_O (3, 12). We found 9 of 40 patients (22.5%) with opening pressure >200 mmH_2_O, two of them above 250 mmH_2_O (12). Each patient with opening pressure >200 repeated the MRV study 1 month after CSF withdrawal to evaluate changes in neuroimaging findings.

Subjects were examined with a 3T MR system (Signa 3T GE) with an eight-channel brain array coil. All the examinations included T1- and T2-weighted sequences and MRV studies. MRV studies were performed using two-dimensional time-of-flight sequence acquired in the coronal plane and by a three-dimensional contrast-enhanced ultrafast gradient-echo angiographic sequence with elliptic centric ordering of k-space collection (Time Resolved Imaging of Contrast Kinetics). The Time Resolved Imaging of Contrast Kinetics sequences acquisition was in the sagittal image plane, covering the whole head. Venous phase correct time delay was calculated administering a small 2-ml test bolus of gadolinium chelates followed by 10 ml bolus of normal saline (12). The angiographic sequence was then acquired after the injection of a 30-ml bolus of gadolinium chelate contrast agent at a rate of 2 ml/s followed by 30 ml saline flush at 2 ml/s (12).

Two neuroradiologists (F.T. and L.C.), who were blinded to the patient's clinical features, reviewed source images from contrast-enhanced MRV on a PACS viewing workstation. Sagittal source images and reformatted images of the volume using 1–2 mm thick section in the coronal and axial plane and three-dimensional maximum intensity projection reconstructions were reviewed. Vascular stenosis was determined using the combined conduit score (CCS) (17). The CCS is defined as the sum of the right and left scores that is the highest degree of stenosis from the torcular to the distal sigmoid sinus, rated on a 0–4 scale as follows: 0, discontinuity; 1, hypoplasia or severe stenosis estimated as <25% of the cross-sectional diameter of the lumen; 2, moderate stenosis (25–50%); 3, mild stenosis (50–75%); and 4, no significant narrowing seen (75–100%) (17). Stenosis was defined as a CCS score <3. The sum of the right and left side scores provided the CCS (17). Stenosis was also defined as bilateral, unilateral, or absent. Disagreements were resolved by consensus. The degree of sinus stenosis was correlated to clinical features including opening pressure, body mass index (BMI), and disease duration.

We evaluated effect of CSF withdrawal at 1, 3, and 6 months follow-up. Patients recorded all headache attacks and drugs treatments on a clinical diary, over the whole study period (12).

### Statistics

All data were analyzed using the SPSS software package (version 21, IBM Analytics). *t*-test or Mann–Whitney test, as appropriate, was used to compare continuous variables, while chi-square test was adopted for categorical variables. Results were expressed by mean ± standard deviation, median with interquartile range or percentage. The Spearman bivariate test was used to detect the strength of correlation between selected variables. Values of *p* < 0.05 were considered statistically significant. A Bonferroni correction was applied for multiple comparisons.

## Results

Analyses were conducted in 40 patients (32 F, 8 M; mean age, 49.4 ± 10.8; mean BMI, 26.7 ± 6.4). Thirty-nine patients had chronic migraine, and one patient had chronic tension-type headache with concomitant episodic migraine. Demographic and baseline clinical features are reported in Table 2. All patients had normal brain parenchyma. Nineteen cases (47.5%) had MRV evidence of transverse sinus stenosis: bilateral in seven patients (17.5%; CCS ranging from 1 to 4) and unilateral in 12 cases (30%, 11 on the left side, and one on the opposite) (CCS ranging from 4 to 6). The remaining 21 MRV studies (52.5%) revealed no transverse sinus stenosis (CCS, 7 or 8) (Table 2). Patients with opening pressure <200 mmH_2_O and those with opening pressure >200 mmH_2_O showed no statistically significant differences in demographic and clinical features, CCS score, and presence or not of unilateral/bilateral stenosis (Table 3). Details on the distribution of transverse sinus stenosis findings according to opening pressure are shown in Table 4. In particular, among the two patients with opening pressure >250 mmH_2_O, one had bilateral sinus stenosis and the other no evidence of stenosis. On Spearman bivariate test, there was no correlation between opening pressure and CCS score. There was no statistically significant difference (*p* = 0.616) in BMI values among groups with absent (mean, 25.85; SD, 5.90), unilateral (mean, 27.92; SD, 7.11), or bilateral (mean, 27.44; SD, 7.29) stenosis. Moreover, no association was detected between BMI and disease duration. No changes of neuroimaging findings and CCS scores were found on neuroimaging in the nine patients with opening pressure >200 mmH_2_O 1 month after CSF withdrawal. At the 1 month follow-up visit, seven of the nine patient reported clinical improvement after withdrawal. Effect of CSF withdrawal on headache up to 6-month follow-up is briefly reported in Table 5 and also in detail elsewhere (12).

**Table 2:** Demographic and baseline clinical characteristics of the study sample.

**Sample**	***N* = 40**
Age (years)	49.4 ± 10.8
**Sex**	
Males	8 (20.0%)
Females	32 (80.0%)
**Marital status**	
Single	7 (17.5%)
Married	27 (62.5%)
Separated/divorced	5 (12.5%)
Widower	1 (2.5%)
**Years of education**	11.3 ± 3.5
**Employment**	
Unemployed	3 (7.5%)
Student	1 (2.5%)
Employee	21 (52.5%)
Housewife	4 (10.0%)
Retired	6 (15.0%)
Self-employed	5 (12.5%)
**BMI**	
<20	2 (5.0%)
20–25	16 (40.0%)
25–30	14 (35.0%)
>30	8 (20.0%)
**Age at headache onset (years)**	16.6 ± 8.3
**Age of headache chronification (years)**	37.8 ± 11.3
**Duration of chronification (years)**	11.6 ± 9.9
**Headache frequency (days/month)**	28.1 ± 4.1
**Frequency of medication intake (days/month)**	26.5 ± 6.9
**Overusers patients**	
Triptans	20 (50.0%)
Simple analgesics and/or NSAIDs	21 (52.4%)
Combination analgesics	15 (37.5%)
**Sinus stenosis**	
Absent (CCS 7–8)	21 (52.5%)
Unilateral (CCS 4–6)	12 (30%)
Bilateral (CCS 1–4)	7 (17.5%)

**Table 3:** Comparisons of features of patients with opening pressure (OP) < 200 mmH_2_O and patients with OP > 200 mmH_2_O (groups 1 and 2).

**Mean value ± SD median (interquartile range) %**	**OP < 200 mmH_**2**_O (*N* = 31)**	**OP > 200 mmH_**2**_O (*N* = 9)**	***p*-values**	**Bonferroni adjusted *p*-values**
Age (years)	49 ± 12	50 ± 8	0.858	n.s.^*^
Female (%)	77.4	88.9	0.776	n.s.^**^
BMI	25 ± 5 24 (22–27)	32 ± 7 34 (25–38)	0.015	n.s.^*^
Opening pressure	159 ± 28 163 (143–184)	245 ± 51 224 (211–258)	<0.001	n.s.^*^
Years of education	11 ± 3 13 (8–13)	11 ± 4 13 (8–13)	0.757	n.s.^*^
Duration of chronification in years	10 ± 9 10 (3–15)	16 ± 13 10 (5–28)	0.407	n.s.^*^
CCS score	6 ± 2 8 (5–8)	5 ± 2 5 (3–7)	0.076	n.s.^*^
Unilateral stenosis	10 (32.3%)	2 (22.2%)	0.869	n.s.^**^
Bilateral stenosis	3 (9.7%)	4 (44.4%)	0.055	n.s.^**^
No stenosis	18 (58.1%)	3 (33.3%)	0.353	n.s.^**^

n.s. not significant;

* p < 0.008 were considered statistically significant;

** *p < 0.01 were considered statistically significant*.

**Table 4:** Distribution of transverse sinus stenosis in our sample, based on opening pressure measurement.

	**Total (*N* = 40)**	**OP < 200 mmH_**2**_O (*N* = 31)**	**200 < OP < 250 mmH_**2**_O (*N* = 7)**	**OP > 250 mmH_**2**_O (*N* = 2)**
Bilateral TSS	7	3	3	1
Unilateral TSS	12	10	2	0
No TSS	21	18	2	1

*TSS, transverse sinus stenosis; OP, CSF opening pressure*.

**Table 5:** Features of patients that underwent cerebrospinal fluid (CSF) withdrawal.

**Patient**	**Headache diagnosis**	**Duration of chronification (years)**	**Headache frequency (days/month)**	**Overused drugs and Frequency of medication intake (days/month)**	**BMI**	**CCS score**	**TSS on MRV**	**OP (mmH_**2**_O)**	**CSF withdrawal (ml)**	**Improvement after CSF withdrawal**	**Duration of improvement (follow-up) (month)**
1	CM	2	25	Triptans (<10)	35.6	2	Bilateral	245	16	EM, Tinnitus	(Got pregnant)
2	CM	9	30	Combination analgesics (30)	35.2	5	Left	224	12	None	–
3	CM	10	30	Triptans (30)	25.4	5	Left	204	18	EM	3
4	CM	28	30	Combination analgesics (30)	25.4	3	Bilateral	218	12	Reduced intensity of headache and drug intake	1
5	CTTH + EM	37	30	Triptans (4)	26.5	4	Bilateral	204	8	None	–
6	CM	28	26	Triptans (20)	43	7	Absent	224	8	EM	6
7	CM	2	30	Triptans (10)	39.5	2	Bilateral	367	14	Reduced intensity of headache	1
8	CM	7	30	Triptans (30)	22.1	8	Absent	245	20	EM	6
9	CM	17	20	Combination analgesics (20)	34.4	8	Absent	272	26	Reduced intensity of headache and drug intake	1

*CM, chronic migraine; CTTH, chronic tension-type headache; EM, episodic migraine; MRV, magnetic resonance venography; TSS, transverse sinus stenosis; OP, CSF opening pressure*.

## Discussion

In our study, we found a relatively high frequency (47.5%) of transverse sinus stenosis in chronic headache (chronic migraine and chronic tension-type headache) patients refractory to preventive treatments. Previously reported transverse sinus stenosis frequency ranges from 9% in a series of 198 chronic tension-type headache patients to 92.8% in 44 unresponsive chronic migraine patients (1–4) (Table 6). Undoubtedly, our findings are significantly lower than previously described in this latter series of refractory chronic migraine patients, but similar to those found in a series of 83 unselected chronic migraine patients (50.6%) (3, 4). These heterogeneous results may in part be explained by a lack of homogeneity in the MRV technique used among studies.

**Table 6:** Summary of previous studies investigating transverse sinus stenosis in chronic headache patients.

**References**	**Diagnosis of chronic headache**	**MRV scanner**	**Number of patients**	**TSS (*N*; %)**	**Bilateral TSS (*N*; %)**	**Unilateral TSS (*N*; %)**
Bono et al. (1)	CTTH	1.5 T	198	18 (9%)	18 (100%)	0 (0%)
Bono et al. (2)	CM, CTTH	0.5–1.5 T	98	65 (66%)	48 (49%)	17 (17%)
De Simone et al. (3)	Refractory CM	Heterogeneous	56	52 (92.8%)	15/44 (34%)	29/44 (66%)
Fofi et al. (4)	CM	1.5 T	83	42 (50.6%)	0 (0%)	42 (100%)

*CM, chronic migraine; CTTH, chronic tension-type headache; MRV, magnetic resonance venography; TSS, transverse sinus stenosis*.

Furthermore, our results confirm that in chronic migraine and chronic tension-type headache patients, independent of opening pressure, transverse sinus stenosis may be bilateral (17.5%) or unilateral (30%). Interestingly, unilateral transverse sinus stenosis prevalence in our sample is in line with the 33% reported in the general population (11). On the contrary, our bilateral transverse sinus stenosis prevalence (17.5%) was significantly higher than the 5% found in healthy subjects (11).

Overall, our results suggest that transverse sinus stenosis is a frequent radiological feature in refractory patients, even though the role is still unknown. We speculate that this neuroimaging feature may be a risk factor for chronic migraine or a comorbidity. On the contrary, this finding alone should not be considered an isolated hallmark of intracranial hypertension in chronic migraine and chronic tension-type headache patients. In fact, we found no correlation between CCS score and CSF opening pressure. Moreover, in our series, transverse sinus stenosis persisted after CSF withdrawal, as previously reported (18). This contrast with recent evidence of the reversibility of stenosis after intracranial pressure normalization (19, 20). We may not exclude that the difference in outcome can be related to different timing of MRV after withdrawal and to the hypothesis that transverse sinus stenosis is only one of the contributing factors involved in intracranial hypertension (9, 12). A recent retrospective study supported this hypothesis, reporting that no individual radiological feature of intracranial hypertension (transverse sinus stenosis, empty sella, optic nerve sheath diameter, and flattening of the posterior sclerae) had sufficient specificity to be diagnostic for raised opening pressure. On the contrary, a combination of any three of these four MRI features was found to be nearly 100% specific and 64% sensitive for intracranial hypertension (21). Consequently, as a corollary, our findings confirm the need to combine opening pressure and neuroradiological criteria to make the diagnosis of IIHWOP.

The strength of this study is that all patients were consecutively evaluated using a 3T MR system with optimal structural details of intracranial venous system. The main limitations of the study include the relatively small sample, the lack of a control group, and the absence of CSF opening pressure measurement at follow-up. Moreover, due to feasibility reasons, patients underwent MRV only once during follow-up, set at 1 month after withdrawal as the first follow-up visit. Future epidemiological or multicenter studies with a larger number of refractory chronic migraine and chronic tension-type headache patients are needed to confirm these results.

## Conclusion

Transverse sinus stenosis is a frequent radiological finding in chronic headache patients, mainly in refractory chronic migraine. Its prevalence in chronic headache sufferers is slightly higher than in the general population. However, isolated unilateral or bilateral transverse sinus stenosis finding is not suggestive of intracranial hypertension. Whether transverse sinus stenosis may be a possible risk factor for chronic headache or a comorbidity needs to be evaluated in larger epidemiological studies.

## Data Availability Statement

The datasets generated for this study are available on request to the corresponding author.

## Ethics Statement

The studies involving human participants were reviewed and approved by the Local Ethics Committee of the local health service of Bologna, Italy (no. 12017/CE). The patients/participants provided their written informed consent to participate in this study.

## Author Contributions

VF, GP, LC, FT, CL, and SC designed the study. VF, GP, LC, FT, CL, MM, RA, and SC acquired the data. SA-R analyzed the data. All authors interpreted the data, contributed to manuscript revision, and read and approved the submitted version. VF and SC drafted the manuscript. PC revised it critically for important intellectual content.

### Conflict of Interest

GP has served on advisory boards for Allergan plc and has received honoraria for speaking engagements or consulting activities with Teva and Allergan plc. CL has received speaker honoraria and travel reimbursement for meetings from Santhera Pharmaceuticals. PC has received honoraria for speaking engagements or consulting activities with Allergan Italia, AbbVie srl, Chiesi Farmaceutici, Teva, UCB Pharma S.p.A., Zambon. SC has received honoraria for speaking engagements or consulting activities with Teva, Novartis. The remaining authors declare that the research was conducted in the absence of any commercial or financial relationships that could be construed as a potential conflict of interest.
